# Does Asthma Affect the Risk of Developing Breast Cancer?

**DOI:** 10.1002/cam4.70539

**Published:** 2024-12-31

**Authors:** Karin B. Michels, Orianne Dumas, Raphaelle Varraso, Carlos A. Camargo

**Affiliations:** ^1^ Institute for Prevention and Cancer Epidemiology, Faculty of Medicine and Medical Center University of Freiburg Freiburg Germany; ^2^ Department of Epidemiology, Fielding School of Public Health University of California Los Angeles California USA; ^3^ Équipe d'Épidémiologie Respiratoire Intégrative, Inserm, CESP, Université Paris‐Saclay, UVSQ, Univ. Paris‐Sud Villejuif France; ^4^ Department of Emergency Medicine, Massachusetts General Hospital Harvard Medical School Boston Massachusetts USA; ^5^ Department of Epidemiology Harvard T. H. Chan School of Public Health Boston Massachusetts USA

## Abstract

**Background:**

The role of the immune system in cancer defense is likely underappreciated. While there has been longstanding interest in the role of atopic diseases in cancer, only a few studies have tested this hypothesis.

**Methods:**

We analyzed data from 202,055 women participating in the Nurses' Health Study (NHS) and the Nurses' Health Study II (NHS II) to explore whether asthma is associated with breast cancer. We used Cox proportional hazards models to link physician‐diagnosed asthma with subsequent incidence of breast cancer.

**Results:**

Across the two cohorts, we identified 18,403 cases of physician‐diagnosed asthma. During 4,393,760 person‐years of follow‐up, 11,096 incident cases of breast cancer were diagnosed. In NHS, women with asthma had a covariate‐adjusted hazard ratio of 0.92 (95% CI: 0.86–0.99) to develop breast cancer compared to women without asthma; the respective HR in NHS II was 0.93 (0.84–1.03), and 0.92 (0.87–0.98) in the pooled analysis. Among never‐smokers, the HR for breast cancer was 0.91 (0.81–1.02) in NHS, 0.81 (0.70–0.93) in NHS II, and 0.86 (0.77–0.97) combined. In two large prospective cohorts of women, participants with asthma had a somewhat lower risk of breast cancer. An active immune system may provide protection from breast cancer.

**Conclusions:**

In these longitudinal studies, women with asthma had a somewhat lower risk of breast cancer. This association was most pronounced among never smokers. An active immune system may provide protection from breast cancer.

## 1 Introduction

Breast cancer is the most common cancer among women [1]. Despite intense research, much of the national and international variation in breast cancer rates still cannot be explained by known or suspected risk factors, including reproductive, anthropometric, and lifestyle factors [2]. One intrinsic factor that may contribute to cancer susceptibility, but that is largely understudied in this context, is the immune system. Most of the interest in the association between immune function and cancer has focused on targeting the immune system to treat, rather than prevent cancer [3]. The scientific premise for the present research is the longstanding interest in the role of atopic diseases—such as asthma, allergic rhinitis (hay fever), and atopic dermatitis (eczema)—in cancer etiology [4].

The hypotheses underlying associations between allergy and cancer are based on two theories. The “antigenic stimulation” theory focuses on atopic disease‐associated chronic inflammation which stimulates cell growth and may support mutations of actively dividing stem cells and malignant proliferation of aberrant cells [5, 6, 7]. The link between chronic inflammation and cancer has been established [8, 9, 10]. The antigenic stimulation hypothesis therefore supports a positive association between atopic disease and cancer.

Conversely, the hypothesis for an inverse association between allergy and cancer is based on enhanced immune function. One hypothesis, the “immune surveillance” theory, is based on the observation that the immune system of individuals with atopic disease recognizes and destroys toxins and foreign particles such as pollen and mold and their hyperactive immune system is similarly able to effectively detect and eradicate autogenic, premalignant cells before tumors develop [11, 12, 13, 14, 15]. Hyperimmunity would therefore provide efficient immune surveillance which becomes useful in clearing the body of premalignant cells and therefore prevents cancer. Another hypothesis that would support an inverse association between allergy and cancer incidence, the “prophylaxis” hypothesis, is based on the allergenic and potential carcinogen avoidance of allergy carriers [16, 17, 18].

There has been interest in the association between atopic disease, specifically asthma, and cancer for decades. Indeed, the field of AllergoOncology has been concerned with defining allergy‐associated biomarkers for cancer [19] and with utilizing the association between allergies and cancer to develop novel therapeutic interventions for both disorders [20, 21]. Studies have considered overall cancer rates [22, 23] or focused on hematological cancers [24, 25], pancreatic cancers [26, 27, 28, 29], glioma [30], colorectal cancers [31, 32], head and neck cancer [33, 34], lung cancer [35], prostate cancer [36], and others. Results varied, with some studies suggesting no association while others suggested a positive or an inverse association. Allergies and atopy have also been related to cardiovascular disease and thrombosis [37].

Fewer studies have evaluated the association between asthma and breast cancer risk and they are limited by the inability to control for potentially important confounding variables, important differences in study design, and lack of stratification to explore effect modification. A 2009 meta‐analysis suggested that women with asthma had a lower risk of breast cancer [38], while one subsequent study failed to find an overall association between asthma and breast cancer risk, however, an inverse association was observed among premenopausal women [39] and in another study a non‐significant inverse association between asthma and breast cancer was observed [22].

The association between asthma and breast cancer incidence has not been explored in a large prospective cohort study that assessed a wealth of covariates, had lengthy follow‐ups, and validated asthma diagnoses. Accordingly, we used data from two large and well‐conducted prospective cohorts of women, the Nurses' Health Study and the Nurses' Health Study II to conduct detailed and well‐controlled analyses of the association between asthma and breast cancer incidence and to consider refined definitions of asthma (as atopic vs. non‐atopic asthma) and to stratify by menopausal status and smoking status.

## 2 Methods

### 2.1 Study Population

We utilized two large cohort studies of women, the Nurses' Health Study (NHS) and the Nurses' Health II (NHS II) to link asthma with the incidence of breast cancer. The Nurses' Health Study was established in 1976 when 121,701 female registered nurses 30–55 years of age completed a mailed questionnaire on their health status and on various potential risk factors for cancer, cardiovascular disease, and other major illnesses. Participants receive follow‐up questionnaires biennially to update information on demographic, anthropometric, and lifestyle factors, and on newly diagnosed diseases, including asthma and breast cancer. For the present analysis we used follow‐up data through 2016. The response rate for NHS remains > 83% after 40 years of follow‐up. The Nurses' Health Study II is an ongoing prospective cohort that was established in 1989 when 116,429 registered nurses from 14 states completed a baseline questionnaire with questions about various demographic and lifestyle factors, anthropometric variables, and disease history. Follow‐up questionnaires are sent biannually to participants with questions updating information on diseases, anthropometric factors, and other risk factors. For the present analysis we used follow‐up data through 2015. Response rates to NHS II questionnaires remain > 78% after 26 years of follow‐up. The study population for the present analysis consisted of 202,055 women participating in either cohort with no history of any cancer before inclusion and with breast cancer diagnosis confirmed by review of medical records (*n* = 96,783 in NHS and 105,272 in NHSII).

The study protocol was approved by the institutional review boards of the Brigham and Women's Hospital and Harvard T.H. Chan School of Public Health, and those of participating registries as required. For both cohorts completion and return of the questionnaires were considered implied consent.

### 2.2 Assessment of Asthma

In NHS, participants were asked starting in 1988 to report whether they had ever been diagnosed with asthma by a physician and, if yes, the year of diagnosis. Asthma cases were identified by biennial self‐reported questionnaires from 1988 onward. Two supplemental asthma questionnaires detailing asthma symptoms, medication use, exacerbation‐related healthcare use, and hayfever/seasonal allergies/allergic rhinitis were sent in 1998 and in 2000 to all participants who reported a physician diagnosis of asthma through 1996.

In NHS II, physician‐diagnosed asthma was asked starting in 1991 and the date of first diagnosis was also assessed (e.g., in 1991: < 1989, 1989–1991, and 1991). We identified asthma cases by biennial self‐reported questionnaires from 1991 onward. Several supplemental asthma questionnaires detailing asthma symptoms, medication use, exacerbation‐related healthcare use, and hayfever/seasonal allergies/allergic rhinitis were sent to NHS II participants to refine asthma definitions in 1993–1995, 1998, 2003, and 2014–2015.

Asthma cases were defined as participants with: (1) physician‐diagnosed asthma from main questionnaires and who confirmed their asthma diagnosis on any supplemental questionnaires and reported using any prescribed long‐term preventive medication (e.g., inhaled corticosteroids) in the past year (from 1988 till 2000 in the NHS and from 1991 till 2013 in the NHSII); (2) or with physician‐diagnosed asthma from main questionnaires after 2000 in the NHS, and after 2013 in the NHSII.

This definition (i.e., confirmed asthma diagnosis and using long‐term preventive medication in the past year) was validated via medical record review in a random sample of nurses from NHSII in 1998. Among 100 randomly selected women with self‐reported incident asthma, 95% had medical record evidence of a physician diagnosis of asthma.

We defined atopic asthma by the concomitant presence of any other atopic disease reported on the supplementary questionnaires. These include hay fever, seasonal allergies, or allergic rhinitis in NHS and NHSII.

In this population, we identified 18,403 cases of physician‐diagnosed asthma (*n* = 7825 in NHS and 10,578 in NHSII), and a subgroup of 14,330 cases of atopic asthma (*n* = 4910 in NHS and 9420 in NHSII).

### 2.3 Assessment of Breast Cancer

On each biennial questionnaire, NHS and NHS II participants were asked whether breast cancer had been diagnosed and, if so, the date of diagnosis. The National Death Index is routinely searched for women who did not respond to the questionnaires. All participants (or next of kin for those who have died) who reported breast cancer were asked for permission to review the relevant medical records and pathology reports for diagnosis confirmation. Medical records were obtained for over 93% of the cases and pathology reports confirmed breast cancer in 99% of women whose reports were reviewed [40]. Due to the high degree of accuracy of the participants' reports among those for whom records were obtained all self‐reported invasive cases of breast cancer were included in the analysis. Cases of carcinoma in situ were censored at the time of diagnosis. Participants were excluded if they had any cancer diagnosed before 1999 or were missing the date of diagnosis of invasive breast cancer.

In NHS, 7498 incident cases of invasive breast cancer were diagnosed between 1988 and 2016 and reported date of diagnosis. In NHS II, 3598 incident invasive breast cancer cases were diagnosed between 1991 and 2015 and reported date of diagnosis. Among the 202,064 women included in the present analyses, 11,096 incident cases of breast cancer were diagnosed during 4,393,760 person‐years of follow‐up.

### 2.4 Covariates

Information on known and suspected risk factors for premenopausal and postmenopausal breast cancer was collected on the baseline and on multiple biennial questionnaires. Participants reported their age, height, history of benign breast disease, family history of breast cancer (in mother, sister, or grandmother), age at menarche, age at first birth, parity, oral contraceptive use, physical activity, alcohol consumption, weight, weight at age 18, weight gain since age 18, menopausal status, and postmenopausal hormone use. Our data file includes a derived menopause variable that takes into consideration natural and artificial cessation of menstrual bleeding. Menopause is defined as 12 months without menstrual bleeding. BMI was calculated as weight in kilograms divided by the square of height in meters. These variables are preselected based on their established or hypothesized associations with breast cancer [41].

### 2.5 Statistical Analysis

In NHS, women who reported prevalent cancer at baseline in 1988 were excluded from the analyses. The NHS II cohort was cancer‐free at baseline in 1989. We used a Cox proportional hazards regression to calculate age‐ and multivariable‐adjusted hazard ratios and 95% confidence intervals of invasive breast cancer by history of asthma at baseline or ever having a diagnosis of asthma during follow‐up (modeled as an updated exposure). Person‐years of follow‐up were calculated as the time from completion of the questionnaire at baseline (1988 for NHS and 1989 for NHS II) and to the date of return of the last available questionnaire (2016 for NHS and 2015 for NHS II), or the date of diagnosis of invasive breast cancer, other cancer, death, or loss to follow‐up, whichever occurred first. During follow‐up, women were censored from the analysis if they developed breast cancer, or any other cancer, if they die, or if they were lost to follow‐up. The proportional hazards model allows us to adjust simultaneously for multiple potential confounders of this association; models were adjusted for age (in months), race (White and Non‐White), smoking (never, former, and current), family history of breast cancer in first degree relative(s) (dichotomous), history of benign breast disease (dichotomous), height (continuous), body mass index (BMI) at age 18 (continuous), current BMI (continuous), age at menarche (≤ 11, 12, 13, 14, and 15+ years), parity (0, 1, 2, 3, and 4+ children), age at first birth (< 25, 25–29.9, 30–34.9, and 35+ years), menopausal status (premenopausal, postmenopausal, and unknown), use of postmenopausal hormones (never, past user, current user, and unknown current status), moderate and vigorous physical activity (NHS: < 3, 3–8.9, 9–17.9, 18–26.9, 27+ METs/week; NHS II:< 3, 3–8.9, 9–17.9, 18–26.9, 27–41.9, and 42+ METSs/week), and alcohol consumption (none, 0.1–5.0, 5.1–10, and 10+ grams per day). Covariate values were updated in the analysis whenever new information was obtained from the biennial questionnaire.

Analyses were conducted separately in each of the two cohorts. We then assessed heterogeneity in the results from the two cohorts. If no significant heterogeneity was present, data were pooled and analyzed using a stratified Cox model including a cohort term.

Effect modification by smoking status and menopausal status was evaluated, and we formally tested for interaction. We stratified by ever cigarette smoking (no and yes), as smoking‐associated asthma tends to be less eosinophilic, and possibly more neutrophilic than asthma not associated with smoking [42]. We also performed analyses stratified by menopausal status since premenopausal and postmenopausal breast cancer have a rather different risk factor profile.

We performed sensitivity analyses to test the robustness of our findings by repeating the analyses using more stringent asthma case definitions, that is, only women who confirmed their asthma diagnosis on any supplemental questionnaires and reported using any prescribed long‐term preventive medication in the past year (i.e., excluding cases identified from main questionnaire only, after 2000 in NHS and after 2013 in NHS II).

## 3 Results

Characteristics of the study populations from NHS and NHS II—according to asthma status—are presented in Tables 1 and 2, respectively. There were no notable differences between women with and without asthma, but there were somewhat less current smokers among women with asthma. Women with asthma were also somewhat less physically active than women without asthma [43].

**TABLE 1:** Age‐standardized characteristics of the study population according to asthma status; Nurses' Health Study, 1988–2016 (*n* = 96,783).

	Asthma		
	No	Yes	Missing (%)
Follow‐up, person‐years	1,943,820	260,980	
Age, mean (SD)^a^	65.6 (10.3)	67.0 (10.1)	0.0
Race			
White	97	97	0.0
Non‐White	3	3	
Smoking habits			
Never smoker, %	45	43	1.2
Former smoker, %	44	49	
Current smoker, %	11	8	
Familial history of breast cancer, %	15	15	0.0
History of benign breast disease, %	17	21	0.1
Height (inches)	64.5 (2.4)	64.5 (2.5)	0.0
BMI at age 18 years (kg/m^2^), mean (SD)	21.3 (2.9)	21.5 (3.1)	12.0
BMI (kg/m^2^), mean (SD)	26.2 (5.2)	27.6 (6.0)	6.0
Age at menarche (year), mean (SD)	12.4 (1.8)	12.4 (1.8)	0.0
Age at menarche			
≤ 11, %	22	25	0.8
12, %	27	26	
13, %	31	30	
14, %	12	12	
≥ 15, %	8	8	
Parity			
Nulliparous, %	5	6	2.3
Number of children in parous women, mean (SD)	2.9 (1.7)	2.8 (1.6)	0.0
Age at first birth, mean (SD)	29.0 (16.7)	29.3 (17.7)	1.6
Menopause status			
Premenopausal, %	7	7	0.1
Postmenopausal, %	90	90	
Dubious menopause, %	3	4	
Use of postmenopausal hormones			
Pre/missing meno, %	7	6	5.4
Never, %	26	19	
Current user, %	28	30	
Past user, %	38	43	
Unknown current status, %	1	1	
Physical activity (METs/w), mean (SD)	18.5 (23.4)	17.1 (22.5)	12.0
Alcohol consumption			15.0
0 g/day, %	42	45	
0.01–5 g/day, %	28	27	
5.01‐10 g/day, %	10	9	
> 10 g/day, %	20	19	

*Note:* Values are means (SD) or percentages and are standardized to the age distribution of the study population. Values of categorical variables may not sum to 100% due to rounding.

Abbreviations: BMI, body mass index; METs/w, metabolic equivalents per week; SD, standard deviation.

^a^ Value is not age‐adjusted.

**TABLE 2:** Age‐standardized characteristics of the study population according to asthma status; Nurses' Health Study II, 1991–2015 (*n* = 105,272).

	Asthma		
	No	Yes	Missing, %
Follow‐up, person‐years	1,954,592	234,368	
Age, mean (SD)^a^	47.2 (8.9)	49.84 (8.5)	0.0
Race			
White, %	96	96	0.0
Non‐White, %	4	4	
Smoking habits			0.1
Never smoker, %	65	66	
Former smoker, %	26	27	
Current smoker, %	9	7	
Familial history of breast cancer, %	11	11	0.0
History of benign breast disease			0.0
No, %	53	46	
Yes‚ unconfirmed, %	28	32	
Yes‚ confirmed, %	19	22	
Height (cm), mean (SD)	164.8 (6.6)	164.6 (6.8)	0.1
BMI at age 18 years (kg/m^2^), mean (SD)	21.2 (3.2)	21.7 (3.8)	0.9
Current BMI (kg/m^2^), mean (SD)	26.4 (6.03)	28.7 (7.37)	4.0
Age at menarche			0.3
≤ 11, %	24	28	
12, %	30	30	
13, %	28	26	
14, %	11	9	
15+, %	8	7	
Parity			0.0
Nulliparous, %	19	22	
Number of children in parous women, mean (SD)	2.3 (0.9)	2.2 (0.9)	0.0
Age at first birth, mean (SD)	26.6 (4.8)	26.5 (5.0)	1.0
Menopause status			6.1
Premenopausal, %	61	60	
Postmenopausal, %	39	40	
Use of postmenopausal hormones			4.5
Never, %	60	53	
Past user only, %	19	22	
Current user, %	20	23	
Past user and unknown current status, %	1	1	
Physical activity (METs/w), mean (SD)	22.8 (28.6)	21.3 (27.7)	13.4
Alcohol consumption			18.5
0 g/day, %	38	42	
0.01–5 g/day, %	34	33	
5.01–10 g/day, %	12	11	
> 10 g/day, %	16	14	

*Note:* Values are means (SD) or percentages and are standardized to the age distribution of the study population. Values of polytomous variables may not sum to 100% due to rounding.

Abbreviations: BMI, body mass index; METs/w, metabolic equivalents per week; SD, standard deviation.

^a^ Value is not age‐adjusted.

Women with asthma were significantly less likely to develop breast cancer among participants of NHS; this association was of borderline significance in NHS II, but statistically significant when data from both cohorts were combined (multivariable hazard ratio [HR] 0.92; 95% confidence interval [CI] 0.87–0.98) (Table 3). No clear difference was evident between atopic and non‐atopic asthma (Table 3). When analyses were stratified by smoking status, the inverse association between asthma and breast cancer incidence was more pronounced among never smokers (multivariable HR 0.86; 95% CI 0.77–0.97) (Table 4). Heterogeneity was observed among ever smokers in the two cohorts (*p* for heterogeneity = 0.03) (Table 4). No important effect modification by menopausal status was observed (Table 5). Among both premenopausal and postmenopausal women, the inverse association between asthma and breast cancer incidence was more pronounced among never smokers (Table 5).

**TABLE 3:** Association between asthma and incident breast cancer among 202,055 participants in the Nurses' Health Studies I and II from 1976 until 2016, and 1989 until 2015, respectively.

	NHS						NHS II						Meta‐analysis	
	Person‐years	No. of cases	Age‐adjusted HR		Multivariable‐adjusted HR		Person‐years	No. of cases	Age‐adjusted HR		Multivariable‐adjusted HR		Multivariable‐adjusted HR	
			HR	95% CI	HR	95% CI			HR	95% CI	HR	95% CI	HR	95% CI
Asthma^a^														
No (ref.)	1,943,820	6669	1	—	1	—	1,954,592	3188	1	—	1	—	1	—
Yes^b^	260,980	829	0.96	0.89–1.03	**0.92**	**0.86–0.99**	234,368	410	0.96	0.87–1.07	0.93	0.84–1.03	**0.92**	**0.87–0.98**
Non‐atopic asthma	27,004	87	0.92	0.75–1.14	0.91	0.73–1.12	18,012	34	1.00	0.71–1.40	1.00	0.71–1.41	0.93	0.78–1.12
Atopic asthma	133,905	444	0.99	0.90–1.09	0.95	0.86–1.04	208,765	363	0.96	0.86–1.07	0.92	0.82–1.02	0.93	0.87–1.01

*Note:* Multivariable models were adjusted for age, race, smoking status, family history of breast cancer in first‐degree relative(s), history of benign breast disease, height, body mass index at age 18, current BMI, age at menarche, parity, age at first birth, menopausal status, use of postmenopausal hormones, moderate and vigorous physical activity, and alcohol consumption. Values with statistical significance at the 0.05‐level are highlighted in bold.

Abbreviations: CI, confidence interval; HR, hazard ratio; NHS, nurses' health study.

^a^ Modeled as an updated exposure during follow‐up.

^b^ Observations with missing values for height were excluded from analyses. Observations with missing values for other variables were included in the model as a “missing” category.

**TABLE 4:** Association between asthma and incident breast cancer among 202,055 participants in the Nurses' Health Studies I and II from 1976 until 2016, and 1989 until 2015, respectively, stratified by smoking status.

	Person‐years	No. of cases	Multivariable‐adjusted HR		*p*
			HR	95% CI	
NHS					
Never‐smokers	990,690	3231	0.91	0.81–1.02	0.10
Ever smokers	1,209,931	4250	0.93	0.85–1.02	0.12
P‐interaction			0.94		
NHS II					
Never‐smokers	1,432,135	2240	**0.81**	**0.70–0.93**	**0.002**
Ever smokers	754,264	1355	1.14	0.97–1.34	0.11
P‐interaction				**0.009**	
Meta‐analysis					
Never‐smokers	2,505,650	5556	**0.86**	**0.77–0.97**	**0.017**
Ever smokers (*p*‐heterogeneity = 0.03)	2,046,318	5709	1.02	0.83–1.24	0.87
P‐interaction			0.15		

*Note:* Multivariable models were adjusted for age, race, family history of breast cancer in first‐degree relative(s), history of benign breast disease, height, body mass index at age 18, current BMI, age at menarche, parity, age at first birth, menopausal status, use of postmenopausal hormones, moderate and vigorous physical activity, and alcohol consumption. Asthma and smoking status were modeled as an updated exposure during follow‐up. Observations with missing values for height were excluded from analyses. Observations with missing values for other variables were included in the model as a “missing” category. Values with statistical significance at the 0.05‐level are highlighted in bold.

Abbreviations: CI, confidence interval; HR, hazard ratio; NHS, Nurses' Health Study.

**TABLE 5:** Association between asthma and incident breast cancer among 202,055 participants in the Nurses' Health Studies I and II from 1976 until 2016, and 1989 until 2015, respectively, stratified by smoking and menopausal status.

	Premenopausal women				Postmenopausal women			
	Person‐years	No. of cases	Multivariable‐adjusted HR		Person‐years	No. of cases	Multivariable‐adjusted HR	
			HR	95% CI			HR	95% CI
NHS, asthma versus no^a^								
All participants	159,404	406	0.98	0.70–1.37	1,974,748	6910	**0.92**	**0.85–0.99**
According to smoking status								
Never‐smokers	77,123	193	0.84	0.50–1.41	880,861	2951	0.91	0.81–1.03
Ever smokers	82,065	212	1.13	0.72–1.77	1,089,980	3943	0.92	0.84–1.02
P‐interaction			0.36				0.98	
NHS II, asthma versus no^a^								
All participants	1,275,717	1699	0.86	0.73–1.03	780,590	1604	0.89	0.77–1.04
According to smoking status								
Never‐smokers	863,193	1099	**0.78**	**0.63–0.98**	483,311	944	**0.77**	**0.63–0.94**
Ever smokers	410,959	597	1.01	0.76–1.35	296,454	660	1.11	0.90–1.38
P‐interaction			0.07				0.09	
Meta‐analysis, asthma versus no^a^								
All participants	1,440,419	2110	0.89	0.76–1.04	2,941,075	8727	**0.91**	**0.86–0.98**
According to smoking status								
Never‐smokers	942,858	1296	**0.79**	**0.64–0.97**	1,445,459	4638	0.85	0.73–1.00
Ever smokers	495,158	809	1.05	0.82–1.33	1,467,037	4705	0.99	0.83–1.18
P‐interaction			0.08				0.22	

*Note:* Multivariable models were adjusted for age, race, family history of breast cancer in first‐degree relative(s), history of benign breast disease, height, body mass index at age 18, current BMI, age at menarche, parity, age at first birth, use of postmenopausal hormones, moderate and vigorous physical activity, and alcohol consumption.

Abbreviations: CI, confidence interval; HR, hazard ratio; NHS, nurses' health study.

^a^ Modeled as an updated exposure during follow‐up. Observations with missing values for height (0.1%) were excluded from analyses (multivariable‐adjusted models). Observations with missing values for other variables were included in the model as a “missing” category. Vales with statistical significance at the 0.05‐level are highlighted in bold.

In sensitivity analyses, restricting the asthma case definition to a validated asthma case definition, results did not appreciably change (Tables S1 and S2).

## 4 Discussion

In these two large prospective cohorts of women, we observed an inverse association between asthma and breast cancer incidence. Results for atopic and non‐atopic asthma did not appreciably differ. Inverse associations were most pronounced among never smokers. No clear effect modification by menopausal status was observed.

Several studies have considered the association between asthma and breast cancer but there is ongoing uncertainty about the nature of this association. Ten studies published between 1985 and 2006 were combined in a 2009 meta‐analysis [38]. Of the ten studies, seven were prospective cohorts and three were retrospective case–control studies. Studies were combined despite the heterogeneity of *I* ^2^ = 91% and yielded an overall relative risk (RR) of 0.81 (95% CI 0.77–0.86) using a fixed effects model and a RR = 0.93 (95% CI (0.73–1.19)) using a random effects model. Excluding the study with the most extreme result (a large cohort) reduced the *I* ^2^ value to 4% with an RR of 0.98 (95% CI 0.91–1.06) for both statistical approaches. Effect estimates for cohort studies and case–control studies combined separately did not appreciably differ [38]. No association was observed between atopy and breast cancer [38]. In a subsequent case–control study conducted in Canada, no association was found between a diagnosis of asthma and breast cancer risk (OR = 0.99; 95% CI 0.85–1.17), however, risk estimates were only adjusted for age [39]. Menopausal status emerged as a statistically significant effect modifier (*p* for interaction = 0.01) with an inverse association among premenopausal women (OR = 0.72; 95% CI 0.54–0.97). In an analysis from the Southern Community Cohort Study comprising 501 cases of breast cancer, women with asthma had an HR = 0.82 (95% CI 0.63–1.05), after adjustment for SES factors, BMI, smoking status, alcohol consumption, physical activity, menopausal status, and family history of cancer [22]. A recent analysis using data from a retrospective cohort study based on electronic health records from OneFlorida did not suggest an association (HR = 1.14; 95%CI 0.93–1.41) [23]. Taken together, consistent with our findings, previous studies seem to suggest a modest inverse association between physician‐diagnosed asthma and breast cancer risk rather than a positive link, promoting the immunosurveillance hypothesis.

Immunoglobulin E (IgE) has been established as a key player in atopic asthma facilitating immune reactions [44]. IgE‐mediated immune reactions may be part of the mechanisms underlying the immunosurveillance against cancer [19, 45, 46]. For example, combined data from four prospective cohorts, including the NHS, suggested an inverse association between borderline elevated IgE levels and glioma (RR = 0.63; 95% CI 0.42–0.93) [30]. Pearce and colleagues estimated that only 20%–40% of asthma has an atopic origin [47]. However, since atopy is often defined by total IgE serum levels, most asthma studies have suggested considerably higher atopy proportions [47]. While cut‐offs for IgE levels to define atopy vary, most individuals with asthma have somewhat elevated IgE levels [47]. It has been suggested that raised IgE levels may in part be a consequence of asthma rather than a cause and marker of autoimmunity [48]. Hence elevated IgE levels may mediate immunosurveillance against cancer in asthma classified as atopic and non‐atopic alike. This may explain why we did not observe any appreciable difference in the association of the two asthma types and breast cancer incidence in our study.

While we did not observe important differences by menopausal status, the inverse associations between a diagnosis of asthma and incident breast cancer were more pronounced among never smokers (Tables 4 and 5). To the best of our knowledge, no prior study was stratified by smoking status. Tobacco smoking suppresses immune function [49] and may thereby negate the immunosurveillance associated with asthma.

Our immune system is increasingly challenged by environmental factors while being compromised by lifestyle factors, such as stress, lack of sleep, poor nutrition, and associated disorders such as obesity. A weakened intrinsic defense system may be permissive to tumor cell growth, replication, and spread. Understanding the role of disorders associated with enhanced immune function (such as asthma) in breast cancer development provides a model to identify opportunities to target the immune system to prevent breast cancer development. As there is currently a dearth of data on the role of such immune markers in breast cancer etiology, our and future studies building upon it may provide new avenues for the understanding of breast cancer mechanisms and may translate into improved opportunities for breast cancer prevention.

The study has potential limitations. The study relied on self‐reported data for both physician‐diagnosed asthma and breast cancer, however, both diagnoses have been validated and showed a very high degree of validity in these cohorts of health professionals. While it would have been preferable to base a diagnosis of atopy on skin prick testing and/or specific serum IgE levels, such data are not available in these large cohorts of more than 200,000 women. Accordingly, we chose a proxy that has been used by other investigators to examine potential differences between atopic versus non‐atopic asthma. Further strengths of our study include the large sample size and high number of cases as well as the long follow‐up and integrity of the cohort with limited loss‐to‐follow‐up. Moreover, we were able to adjust for a considerable number of covariates and had sufficient power to perform stratified analyses.

In summary, our study provides evidence for the immunosurveillance hypothesis linking asthma with a reduced incidence of breast cancer. Our results support the important role of the immune system for breast cancer development, a mechanism that has been underappreciated so far.

## Author Contributions

**Karin B. Michels:** conceptualization (lead), data curation (equal), methodology (lead), project administration (equal), writing – original draft (lead), writing – review and editing (equal). **Orianne Dumas:** formal analysis (equal), writing – review and editing (equal). **Raphaelle Varraso:** formal analysis (equal), writing – review and editing (equal). **Carlos A. Camargo Jr:** conceptualization (equal), methodology (equal), project administration (equal), supervision (equal), writing – review and editing (equal).

## Ethics Statement

The study protocol was approved by the institutional review boards of the Brigham and Women's Hospital and Harvard T.H. Chan School of Public Health, and those of participating registries as required. Completion and return of the questionnaires were considered implied consent.

## Conflicts of Interest

The authors declare no conflicts of interest.

## Supporting information


**Data S1 and S2.** Supporting Information.

## Data Availability

Data will be made available by the authors upon reasonable request.
