# Supplementary material for: Does Asthma Affect the Risk of Developing Breast Cancer?
Source: Cancer Med. 2024 Dec 31;14(1):e70539. doi: 10.1002/cam4.70539 (PMC11686484; doi:10.1002/cam4.70539)
Supplement: Supplementary file 1 — Data S1 and S2. Supporting Information. [file CAM4-14-e70539-s001.docx]

| **Supplemental Table 1**. Association between asthma and incident breast cancer among 202,055 participants in the Nurses’ Health Studies I and II from 1976 until 2016, and 1989 until 2015, respectively, restricted to validated asthma diagnosis. | | | | | | | | | | | | | | | | |
| --- | --- | --- | --- | --- | --- | --- | --- | --- | --- | --- | --- | --- | --- | --- | --- | --- |
|  | NHS | | | | | |  | NHS II | | | | | |  | Meta-analysis | |
|  | Person-years | No. of cases | Age-adjusted HR | | Multivariable-adjusted HR | |  | Person-years | No. of cases | Age-adjusted HR | | Multivariable-adjusted HR | |  | Multivariable-adjusted HR | |
|  |  |  | HR | 95% CI | HR | 95% CI |  |  |  | HR | 95% CI | HR | 95% CI |  | HR | 95% CI |
| Asthma |  |  |  |  |  |  |  |  |  |  |  |  |  |  |  |  |
| No (ref.) | 1,971,680 | 6,751 | 1 | - | 1 | - |  | 1,948,525 | 3,178 | 1 | - | 1 | - |  | 1 | - |
| Yes† | 233,120 | 747 | 0.95 | 0.88-1.03 | **0.91** | **0.84-0.98** |  | 232,733 | 409 | 0.97 | 0.87-1.07 | 0.93 | 0.84-1.03 |  | **0.92** | **0.86-0.98** |

Abbreviations: NHS, Nurses’ Health Study; HR, hazard ratio; CI, confidence interval.

Multivariable models were adjusted for age, race, smoking status, family history of breast cancer in first degree relative(s), history of benign breast disease, height, body mass index at age 18, current BMI, age at menarche, parity, age at first birth, menopausal status, use of postmenopausal hormones, moderate and vigorous physical activity, and alcohol consumption.

* Modeled as an updated exposure during follow-up

† Reiterated on at least one of the asthma supplementary asthma questionnaires that a physician had diagnosed her as having asthma, and reported use of asthma medication in the past year. Observations with missing values for height (0.1%) were excluded from analyses (multivariable-adjusted models). Observations with missing value for other variables were included in the model as a “missing” category.

| **Supplemental Table 2**. Association between asthma and incident breast cancer among 202,055 participants in the Nurses’ Health Studies I and II from 1976 until 2016, and 1989 until 2015, respectively, restricted to validated asthma diagnosis and stratified by smoking status. | | | | | | | |
| --- | --- | --- | --- | --- | --- | --- | --- |
|  | Person-years | No. of cases | Multivariable-adjusted HR | | | P |  |
|  |  |  | HR | 95% CI | |  |  |
| NHS |  |  |  |  | |  |  |
| Never-smokers | 990,690 | 3,231 | 0.91 | 0.81-1.03 | | 0.14 |  |
| Ever smokers | 1,209,931 | 4,250 | 0.91 | 0.82-1.00 | | 0.06 |  |
| *P-interaction* |  |  | *0.83* | | | |  |
| NHS II |  |  |  | | | |  |
| Never-smokers | 1,427,267 | 2,231 | **0.81** | | **0.70-0.93** | **0.003** |  |
| Ever smokers | 751,440 | 1,353 | 1.14 | | 0.97-1.34 | 0.10 |  |
| *P-interaction* |  |  | ***0.008*** | | | |  |
| Meta-analysis |  |  |  | |  |  |  |
| Never-smokers | 2,500,782 | 5,547 | **0.86** | | **0.77-0.97** | **0.02** |  |
| Ever smokers (p-heterogeneity=0.02) | 2,043,494 | 5,707 | 1.01 | | 0.80-1.26 | 0.93 |  |
| *P-interaction* |  |  | *0.28* | | | |  |
| Abbreviations: HR, hazard ratio; CI, confidence interval; NHS, Nurses’ Health Study  Multivariable models were adjusted for age, race, family history of breast cancer in first degree relative(s), history of benign breast disease, height, body mass index at age 18, current BMI, age at menarche, parity, age at first birth, menopausal status, use of postmenopausal hormones, moderate and vigorous physical activity, and alcohol consumption.  Asthma (confirmed in supplemental questionnaire) and smoking status were modeled as an updated exposure during follow-up.  Observations with missing values for height (0.1%) were excluded from analyses. Observations with missing value for other variables were included in the model as a “missing” category. | | | | | | | |
